# Delayed presentation, diagnosis, and psychosocial aspects of male breast cancer

**DOI:** 10.1002/cam4.2953

**Published:** 2020-03-13

**Authors:** Michael Co, Andrea Lee, Ava Kwong

**Affiliations:** ^1^ Division of Breast Surgery The University of Hong Kong Hong Kong; ^2^ Division of Breast Surgery The University of Hong Kong Shenzhen Hospital Shenzhen China; ^3^ Department of Surgery Queen Mary Hospital Hong Kong; ^4^ Hong Kong Hereditary Breast Cancer Family Registry Hong Kong; ^5^ Hong Kong Sanatorium and Hospital Hong Kong

## Abstract

**Introduction:**

Male breast cancer is uncommon, delay in seeking medical attention often results in late presentation and poor prognosis.

**Methods:**

Retrospective review of a prospectively maintained database was performed. Patients who were still having regular follow‐up were contacted for telephone interview.

**Results:**

In this study, 56 male breast cancer patients were treated in our center from January 1998 to December 2018, accounting for 0.88% of all breast cancers treated during the same period of time. Median age of onset was 61 years old (Range: 33‐95). In this study, 6 (10.7%) patients presented with distant metastasis at the time of diagnosis and received palliative systemic treatment only. And, 50 patients were surgically treated and all had mastectomy. Axillary dissection was performed in 36 (72%) patients, while sentinel node biopsy was performed in 14 (28%) patients. Median tumor size was 23 mm (2‐100 mm). A Majority were diagnosed with invasive carcinoma (NOS), while 38 (67.8%) patients were node positive.Here 36 (64.3%) patients were alive at the time of the study, 31 (86.1%) patients responded to the telephone interview. More than 90% of our patients expressed various degrees of embarrassment at the time of breast symptom onset. Similarly, more than 90% of these patients experienced embarrassment while waiting in the breast center with predominant female patients. Most patients (N = 26) were not aware that breast cancer can occur in men prior to the diagnosis. Median duration from symptoms to the first medical consultation was 12.4 months (1‐120 months).

**Conclusion:**

Male breast cancer is rare and patients usually present late, Lack of knowledge, public education, and embarrassment are the important related factors.

## Graphical abstract

Delayed presentation to medical care is common in male breast cancers, resulting in late presentation and poor prognosis. Reasons for the delayed presentation include lack of awareness of male breast cancer and embarrassment to feminine symptoms.

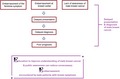

## 1 INTRODUCTION

Breast cancer is often regarded as a female disease. However, breast cancers in men are not as uncommon as perceived. They comprise 1% of all breast cancer, with rising incidence.^1^ Unfortunately, male breast cancer is often identified late, with lymph node involvement and advanced staging on diagnosis, hence worse prognosis.^2^ When compared to female counterparts, behavior of breast cancer in men is actually similar. They present with a palpable mass, harbor the same types of tumor histology, and respond to similar treatment. In fact, when matched for age and staging, men fare comparably to women in outcome and survival.

Since breast cancer behaves similarly in both sexes, the diagnostic delay in men should be addressed. Literature search of this topic yields mostly case series studying the clinicopathological features and treatment approach to the disease. Not many have investigated the reasons for late diagnosis – this paper aims to study this issue.

## 2 METHODS

Institutional review board approval with written informed consent was obtained for personal data collection, analysis and publication. A retrospective review of a prospectively maintained database was performed to identify all male breast cancer patients treated between January 1998 and December 2018. Patient demographic data and clinicopathological characteristics were summarized. Duration of symptom onset to first medical consultation was retrieved and analyzed.

**Figure 1:**
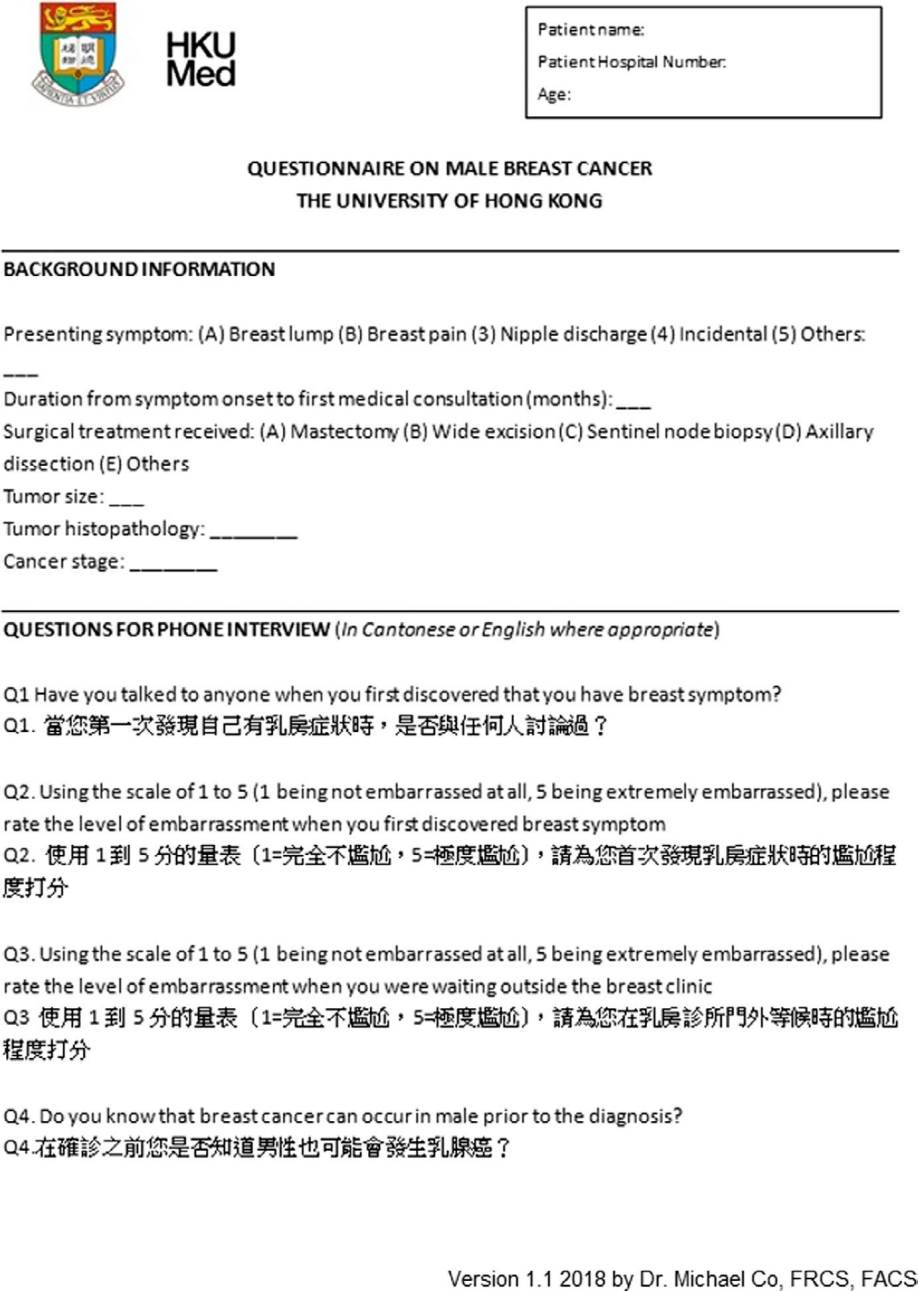
Questionnaire used for the phone interview, interview was done in Cantonese or English (Official languages in Hong Kong)

Male breast cancer survivors at the time of the study were contacted for phone interview in December 2018 with additional verbal consent. All phone interviews were conducted by a single investigator to avoid inter‐interviewer variation in terms of the language used. A standardized questionnaire was used for the phone interview, which contains four questions. Question one was on the level of embarrassment for the breast symptom, question two was on the level of embarrassment for waiting in the waiting outside the breast clinic, question three was the first person to spoke to when patient first discovered breast symptom, question four was whether or not that patient is aware of male breast cancer prior to the diagnosis. (Please refer to Figure 1 for the questionnaire template).

## 3 RESULTS

From January 1998 to December 2018, there were 56 male breast cancer patients treated in our center, accounting for 0.88% of all breast cancers treated during the same period of time. Median age of onset was 61 years old (Range: 33‐95), 9 (16.1%)

patients had a positive family history of breast cancer, of which 7 (77.8%) was first‐degree relatives and 55 (98.2%) patients were presented initially with a painless breast lump, 1 (1.8%) patient had incidental finding of breast lump by a general practitioner during chest auscultation. Mean duration from symptom onset to first consultation was 12.4 months (Range 1‐120 months).

Core needle biopsy was performed in all 56 patients. There were 44 (78.6%) invasive ductal carcinoma, 5 (8.9%) ductal carcinoma in situ (DCIS), 1 (1.8%) invasive lobular carcinoma, and 6 (10.7) patients with other cancer histotypes.

In this study, 6 (10.7%) patients presented with distant metastasis at the time of diagnosis and received palliative systemic treatment only. All the remaining 50 patients received operation with mastectomy. Axillary dissection was done in 36 (72%) patients, while sentinel node biopsy was performed in 14 (28%) patients. Median tumor size was 23 mm (2‐100 mm); 38 (67.8%) patients were node positive.

36 (64.3%) patients were alive at the time of the study, 31 (86.1%) patients responded to telephone interviews and. 18 (58%) patients had experienced “very” to “extremely” severe embarrassment (Likert score of 4 to 5 out of 5) at the time symptom onset, while 11 (35.5%) patients experienced mild to moderate embarrassment (Likert score 2‐3). Only 2 (6.5%) experienced no embarrassment at all. (Table 1).

**Table 1:** Level of embarrassment at the time of symptom and at the waiting area outside breast clinic, in Likert scale of 1‐5

Level of embarrassment	Feel embarrassed at time of symptom *Number of patients*	Feel embarrassed at breast clinic *Number of patients*
5 (Extremely embarrassed)	8 (25.8%)	7 (22.6%)
4 (Very embarrassed)	10 (32.3%)	9 (29.1%)
3 (Moderately embarrassed)	5 (16.1%)	5 (16.1%)
2 (Mildly embarrassed)	6 (19.4%)	8 (25.8%)
1 (Not embarrassed at all)	2 (6.4%)	2 (6.4%)

Similarly, 16 (51.6%) patients experienced "extreme" or "very" severe embarrassment (Likert score 4 to 5 out of 5) while waiting in the clinic among other female patients. 13 (41.9%) patients experienced "mild" to "moderate" level of embarrassment (Likert score 2 to 3). Only 2 (6.5%) experienced no embarrassment at all. (Table 1).

19 (61.3%) patients disclosed and discussed with their spouses when they first discovered the breast symptom, 3 (9.7%) patients discussed with friends while 1 (3.2%) patient discussed with sibling. Of note, 8 (25.8%) patients had never talked to anyone else prior to the medical consultation.

In addition, most patients (N = 26, 83.9%) were not aware that breast cancer can occur in men before the diagnosis.

## 4 DISCUSSION

The incidence of male breast cancer continues to rise. Despite similar presentation and treatment strategies between breast cancer in both sexes, men fare infamously worse, with reported 5‐year survival rates of only 40%‐65%.^1^, ^2^ The main reason for this, as reported in our data and numerous others’, is advanced staging on diagnosis. In our study, 67.8% had lymph node metastasis on presentation, with 10.7% distant metastasis at the time of diagnosis. Mean time taken from symptom onset to diagnosis was 1 year with longest delay of up to 10 years in the current cohort. Our results were consistent with the current literature; delay of seeking medical attention varies from 14‐21 months.^3^ We believe that such delays in diagnosis are preventable. Interviews of our male breast cancer patients explored three key issues contributing to late presentations – lack of awareness, embarrassment, and the need for social support.

Breast cancer in men is often overlooked as it identified and perceived as a female disease. The initiation of pink ribbon culture since the early 1990’s has successfully aroused breast cancer awareness. However, the term “breasts” and the color “pink” are traditionally associated with femininity. Women nowadays have increased breast cancer awareness, and are referred promptly for investigation of any breast mass. Although male breast cancer often presents similarly with a lump, men fail to seek attention early due lack of awareness.^4^, ^5^ Even if a lump is found, it has been suggested that men are more likely to dismiss the pathology as there is no associated pain^5^ and they view breasts merely as a vestigial anatomy.^5^ Studies have shown that such lack of awareness persists at both public and professional levels.^7^ Some men reported that their symptoms were initially disregarded by primary health care providers, causing ultimate delay in diagnosis.^5^, ^6^, ^8^ In this study conducted in Hong Kong, we did not find any delay in referral to specialist care.

Embarrassment is another prevailing problem that male breast cancer patients face. To many of these men, “breasts” carry a negative connotation when mentioned in the context of a male body.^9^ In an American interview study looking into the awareness of male breast cancer in 28 male individuals, one participant stated that “men don't have breasts, they have chests.”^4^ About 43% of participants reported that diagnosis of breast cancer might cause them to question their masculinity.^4^ These sentiments of demasculinization, altered body image, and embarrassment associated with diagnosis of breast cancer in men are observed in several other studies.^10^, ^11^ In a cross‐sectional questionnaire in the UK ,161 male breast cancer patients, 23% of men reported high levels of cancer‐specific distress.^10^ This distress was caused by feelings of altered body image, anxiety, and avoidance coping.^10^ More than 90% of our patients experienced various degree of embarrassment at the time of breast symptom onset. All these individuals admitted that these negative feelings led to hesitation in seeking medical attention. In fact, feelings of embarrassment persisted in the waiting rooms. More than 90% of our patients reported feeling uneasy sitting in the waiting halls surrounded by other patients who were strictly female. A few of these men noted that they were assumed to be the chaperones accompanying wives rather than patients themselves.

Psychosocial and emotional support is crucial to these marginalized men. Gillon writes that men are reluctant to seek medical attention as help‐seeking implies vulnerability and weakness.^12^ In general, research suggests that men tend to turn to women for intimacy and emotional support.^6^, ^13^ In our study, the majority of patients relied on their spouses for disclosure and support, although a significant proportion of our male breast cancer patients did not discuss with any person when they first discovered abnormal breast symptoms.

Proactive partners encouraged men to seek help early, while good spousal relationship provided good psychosocial support. A retrospective study examining the impact of marital status on male breast cancer found that unmarried males were more likely to present with late stage disease and a higher risk of breast cancer‐related death.^14^ Spousal support has been suggested to reduce risk of depression and improve compliance to cancer treatment.^15^

### 4.1 Clinical implications

Due to low incidence of male breast cancer, there is a paucity of knowledge and social support for these patients ^16^, ^17^, ^18^. Of utmost importance is the development of proper media initiative to increase public understanding of this disease^19^. Adequate psychosocial support should be provided to male breast cancer patients. Awareness among primary health care providers also needs to be ensured, in order to avoid dismissal of potentially red‐flag signs of male breast cancer^20^, ^21^. Improvements should be made in creating gender‐specific information. Current breast cancer pamphlets are geared toward women – decorated in pink, illustrated with female anatomy, and doused with advice on breast reconstructive options. Photos of postmastectomy males and information on side effects of hormonal therapy are thought to be important to male breast cancer patients too^11^ – it is the role of health care professionals to readily provide such information to men in need^22^. The gap between men and this neglected disease must be bridged in order to allow earlier diagnosis, prompt treatment, and better prognosis for male breast cancer.

### 4.2 Study limitations

We recognize the inherent limitations of this study of being retrospective in nature and the relatively small case number. However, given that male breast cancer is a truly uncommon disease; our study represents one of the largest single institution cohorts of male breast cancers^23^, ^24^, ^25^, and is one of the few studies on psychosocial aspects of male breast cancers. We believe public breast cancer awareness should cover the male audience, but not just females alone. Access to psychosocial care to male breast cancer patients is as important as female patients.

## 5 CONCLUSION

Male breast cancer is rare and patients usually present late, in which lack of knowledge, public education, and embarrassment are important reasons for the delay in seeking medical attention. Improved psychosocial support to male breast cancer patients is crucial.

## CONFLICT OF INTEREST

All authors report no conflict of interests.

## AUTHOR CONTRIBUTIONS

Michael Co – Conceptualization, data collection, data analysis, manuscript preparation and editing. Andrea Lee – Data collection, manuscript preparation. Ava Kwong – Manuscript revision, final editing and proofreading.

## DISCLAIMERS

Abstract of this study was presented in World Congress of Surgery 2019, Krakow, Poland. Full manuscript has never been submitted elsewhere for publication. This study is not funded by external source.

## Data Availability

The data that support the findings of this study are available on request from the corresponding author. The data are not publicly available due to privacy or ethical restrictions.
